# *In Silico* Screening of the Human Gut Metaproteome Identifies Th17-Promoting Peptides Encrypted in Proteins of Commensal Bacteria

**DOI:** 10.3389/fmicb.2017.01726

**Published:** 2017-09-08

**Authors:** Claudio Hidalgo-Cantabrana, Marco A. Moro-García, Aitor Blanco-Míguez, Florentino Fdez-Riverola, Anália Lourenço, Rebeca Alonso-Arias, Borja Sánchez

**Affiliations:** ^1^Department of Microbiology and Biochemistry of Dairy Products, Instituto de Productos Lácteos de Asturias, Consejo Superior de Investigaciones Científicas Villaviciosa, Spain; ^2^Department of Immunology, Hospital Universitario Central de Asturias Oviedo, Spain; ^3^Escuela Superior de Ingeniería Informática – Department of Computer Science, University of Vigo Vigo, Spain; ^4^Centro de Investigaciones Biomédicas, University of Vigo Vigo, Spain; ^5^Centre of Biological Engineering, University of Minho Braga, Portugal

**Keywords:** bacterial peptides, Th17 response, CD4 cytokines, flow cytometry, microbiome, gut metaproteome

## Abstract

Scientific studies focused on the role of the human microbiome over human health have generated billions of gigabits of genetic information during the last decade. Nowadays integration of all this information in public databases and development of pipelines allowing us to biotechnologically exploit this information are urgently needed. Prediction of the potential bioactivity of the products encoded by the human gut microbiome, or metaproteome, is the first step for identifying proteins responsible for the molecular interaction between microorganisms and the immune system. We have recently published the Mechanism of Action of the Human Microbiome (MAHMI) database (http://www.mahmi.org), conceived as a resource compiling peptide sequences with a potential immunomodulatory activity. Fifteen out of the 300 hundred million peptides contained in the MAHMI database were synthesized. These peptides were identified as being encrypted in proteins produced by gut microbiota members, they do not contain cleavage points for the major intestinal endoproteases and displayed high probability to have immunomodulatory bioactivity. The bacterial peptides FR-16 and LR-17 encrypted in proteins from *Bifidobacterium longum* DJ010A and *Bifidobacterium fragilis* YCH46 respectively, showed the higher immune modulation capability over human peripheral blood mononuclear cells. Both peptides modulated the immune response toward increases in the Th17 and decreases in the Th1 cell response, together with an induction of IL-22 production. These results strongly suggest the combined use of bioinformatics and *in vitro* tools as a first stage in the screening of bioactive peptides encrypted in the human gut metaproteome.

## Introduction

The composition of the human microbiome has revealed strong associations with human health. Alterations in the intestinal microbial populations when compared to control groups, denominated dysbiosis, are nowadays associated with several diseases from intestinal and extraintestinal nature (Shreiner et al., 2015). Modulation of the human microbiome in order to balance the microbial populations toward a healthy situation is a promising intervention to improve human health in the framework of many diseases (Drew, 2016).

The human microbiome performs key metabolic roles such as production of vitamins, short-chain fatty acids and other essential compounds, digestion of several components of the diet such as fibers, gut homeostasis maintenance and modulation of the host immune response (O’Toole et al., 2017). Gut microbiota and human cells communicate with each other through surface-associate proteins or extracellular components, in a dialog denominated molecular cross-talk (Hevia et al., 2015). Moreover, bacteria structural components have their own receptors in the innate immune system; in this way, the constant fraction of the surface-associated flagellin is recognized by Toll-like receptor 5 (TLR-5) and the cell-wall component lipopolysaccharide by TLR-4 (Sanchez et al., 2011). These ligands are denominated microbial associated molecular patterns (MAMPs) and their recognizing counterparts on the human host are termed pattern recognition receptors (PRRs). Recognition and binding of MAMPs to PRRs triggers different intracellular signalization pathways that led to changes on the surface molecules of the immune cells and in their effector secretion profiles, among interleukins and other cytokines (Ley et al., 2006). The type, amount and temporal pattern of MAMP-PRR interaction will finally determine the type of immune response that can be roughly divided into proinflammatory (mainly Th1, Th2 and Th17) or regulatory responses (Treg) (Ruiz et al., 2016a).

Extracellular and surface-associated proteins of the gut microbiota have been studied in the last years as candidates responsible for part of the immunomodulatory action of intestinal microorganisms, given that these proteins performs important roles in the interaction with the host and the environment (Ruiz et al., 2016b). Many of this research has been focused on the identification of molecules responsible for the expansion of Treg cells, a Tcell subset essential in the maintenance of a controlled and low-grade inflammation status of the gut mucosa (Levy et al., 2017). Few proteins involved in gut homeostasis maintenance and produced by gut bacteria have been identified so far. *Faecalibacterium prausnitzii* is a bacterium whose absence from the human gut microbiome is associated with Crohn’s Disease, and which produces a small protein with anti-inflammatory properties by inhibiting the proinflammatory NF-κB pathway (Quevrain et al., 2016). Lactic acid bacteria are transitory members of the human gut microbiota in individuals with fermented product intake within their diets. It has been shown that some lactobacilli species are able to produce immunomodulatory peptides encrypted in larger extracellular proteins. One of these peptides is STp, which when recognized by dendritic cells isolated from Ulcerative Colitis patients induced a switch toward an anti-inflammatory cytokine profile (Bernardo et al., 2012; Al-Hassi et al., 2014).

Therefore, the protein content of an intestinal microorganism may play a key role in the way it modulates the immune system functions in the host (Furusawa et al., 2015). Identifying the molecules responsible for all the molecular crosstalk mechanisms as well as the immune receptors/signalization pathways induced/repressed by the different MAMPs are needed for understanding the immune mechanism of action of a gut microbiota taken as a whole. With these limitations in mind, our research group has developed the Mechanism of Action of the Human Microbiome (MAHMI) database^1^ (Blanco-Miguez et al., 2017). MAHMI is an online resource for the prediction of peptide bioactivity based on their amino acid sequence which is fuelled by public Metahit project metagenomes^2^. This database is a unique resource for the organization and processing of potential immunomodulatory peptides encrypted in the human gut metaproteome.

In this work, we have identified 15 potential bioactive peptides through MAHMI pipeline, to test whether this database was able to identify potential immunomodulatory peptides. The *in silico* test was completed with an *in vitro* test where 15 peptides were incubated with human peripheral blood mononuclear cells (PBMCs), and coupled to a multiplex secreted cytokine assay. Main results are discussed next.

## Materials and Methods

Ethics approval for this study (reference code AGL2013-44039-R) was obtained from the Regional Ethics Committee for Clinical Research (*Comité de Ética de la Investigación del Principado de Asturias*) in compliance with the Declaration of Helsinki. Samples used in this study were obtained from anonymous donors of our blood donation system.

### Bioactivity Prediction

The bacterial peptides used in this study were previously selected regarding their theoretical bioactivity based on *in silico* analyses performed through the MAHMI database^1^ (Blanco-Miguez et al., 2017). Briefly, the pipeline starts with the *in silico* digestion of the proteins obtained from the human gut microbiome (gut metaproteome). Potential bioactivity of the encrypted peptides is performed by comparison of their amino acid sequence to a curated database of immunomodulatory peptides. Selected peptides (**Table 1**) were synthesized at GeneCust facilities (Ellange, Luxemburg). Peptides were resuspended in phosphate buffered saline (PBS) (Oxoid Limited, Hampshire, United Kingdom) at a concentration of 2 mg mL^-1^ and stored at -80°C until their use.

**Table 1:** Potential immunomodulatory peptides used in this work and information associated.

Peptide	Peptide sequence	Uniprot ID	Uniprot protein	Uniprot organism	Uniprot gene	Bioactivity prediction
QE14	QHVENNKSTPLPME	P54355	ENTM_BACFG Fragilysin	*Bacteroides fragilis*	btfP	60
LR17	LPLAFFVLTFLWALILR	Q3V815	MTGA_BACFR Monofunctional biosynthetic peptidoglycan transglycosylase	*Bacteroides fragilis* YCH46	mtgA	80
DW15	DEILENVDEVLGNDW	Q45119	TNPA_BACFG Transposase for insertion sequence element IS21-like	*Bacteroides fragilis*	tnpA	75
KF14	KETILEPVEGVGHF	Q08425	TETQ_BACFG Tetracycline resistance protein TetQ	*Bacteroides fragilis*	tetQ	66
KE13	KKMTPANEARPIE	D4QFE7	BGAL_BIFBI Beta-galactosidase BbgII	*Bifidobacterium bifidum*	bbgII	75
SW14	SASLVDGIARKDPW	D4QFE7	BGAL_BIFBI Beta-galactosidase BbgII	*Bifidobacterium bifidum*	bbgII	66
EY12	EMVEDILTGQCY	C6H178	BGAL2_LACAI Beta-galactosidase LacA	*Lactobacillus acidophilus*	lacA	60
GR16	GTSFEGNEYFEGPSIR	P48790	XYLA_CLOSR Xylosidase/arabinosidase	*Clostridium stercorarium*	xylA	100
AR15	AIQDEIDQLSTEIDR	P80583	FLA_CLOTY Flagellin (Fragment)	*Clostridium tyrobutyricum*	fla	75
FR16	FAIVDEVDSILIDEAR	B3DR89	SECA_BIFLD Protein translocase subunit SecA	*Bifidobacterium longum* DJO10A	secA	100
WK19	WIEAVGYSLTQHPDPELEK	E8MGH8	HYBA1_BIFL2 Non-reducing end beta-L-arabinofuranosidase	*Bifidobacterium longum subsp. longum* ATCC 15707	hypBA1	100
VF10	VPSEKKAELF	P38059	SLAP_LACHE S-layer protein	*Lactobacillus helveticus*	slpH	80
HK11	HIGVHSDPVDK	P30269	AMY_BUTFI Alpha-amylase	*Butyrivibrio fibrisolvens*	amyA	75
AF16	ASPEDEAASMEKAKAF	A6L1Z2	G1095_BACV8 Glycosyl hydrolase family 109 protein 5	*Bacteroides vulgatus* ATCC 8482	BVU_2041	80
NR15	NAALASYSLASDLDR	Q9XD84	TIBA_ECOH1 Adhesin/invasin TibA autotransporter	*Escherichia coli O78:H11* (H10407)	tibA	100

### Peripheral Blood Mononuclear Cells (PBMC) Isolation

The capability of synthetic bacterial peptides to induce immune modulation *in vitro* was assessed using a PBMC model. PBMCs were isolated from the buffy coat of 5 healthy donors from the Community Center for Blood and Tissues of Asturias (Oviedo, Spain). Then, 5 mL of each buffy were diluted with one volume of PBS and added on the top of a 5 mL of Ficoll-Hypaque (Lymphoprep; Nycomed, Oslo, Norway) for gradient separation. Cells were separated by centrifugation (1,800 rpm, 30 min) with two further washes with PBS; the first one at 1,200 rpm, 10 min (to discard platelets) and the second one at 1,500 rpm, 5 min. The number of PBMCs was estimated using a Neubauer chamber (Brand, VWRI Eurolab, Barcelona, Spain) and adjusted to a final concentration of 2 × 106 mL^-1^ in RPMI 1640 broth (Lonza, Basilea, Switzerland) supplemented with 10% (v/v) fetal bovine serum and antibiotics (Sigma-Aldrich, San Luis, MO, United States).

### Co-cultivation of Peptides and PBMCs

Peripheral blood mononuclear cells were cultivated in round bottom 96 wells microplates using 200 μL of the cell suspension described above. Bacterial peptides were added at a final concentration of 50 μg mL^-1^, based on previous studies of the immunomodulatory peptide STp (Bernardo et al., 2012). PBMCs were activated with 100 ng mL^-1^ of anti-CD3 added to the RPMI media. For each donor positive (LPS, 1 μg mL^-1^) and negative controls of PBMCs stimulation were included. Microplates were incubated for 5 days at 37°C with 5% CO_2_.

### Cytokine Quantification

After 5 days, supernatant was collected and stored at -80°C for multiplexed cytokine analyses. The production of 18 different cytokines (GM-CSF, IFNγ, IL-1β, Il-2, IL-4, IL-5, IL-6, IL-9, IL-10, IL-12p70, IL-13, IL-17A, IL-18, IL-21, IL-22, IL-23, IL-27, and TNFα) were quantified using the Th1/Th2/Th9/Th17/Th22/Treg Cytokine 18-Plex Human ProcartaPlex^TM^ Panel (Affymetrix eBioscience, San Diego, CA, United States) and the Luminex^®^ xMap Technology equipment following manufacturer’s settings. The results for each cytokine were represented using box plot diagrams and differences between peptides were statistically analyzed.

**FIGURE 1:**
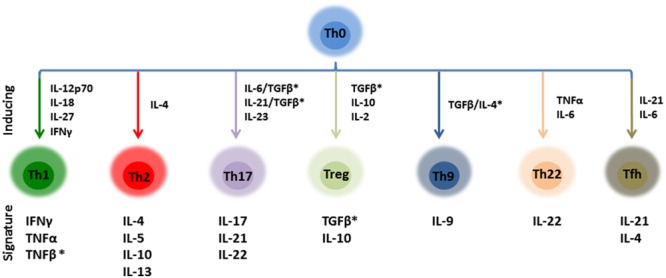
Schematic representation of the main CD4+ effector T cells and their inducing/signature cytokines. Ratios calculated in this work were calculated taken into account this distribution. ^∗^These cytokines were not included in the ratios as TGFβ and TNFβ have not been quantified.

In the case of peptides FR-16 and LR-17, the following ratios were calculated for each sample: IL10/TNFα, IL10/IFNγ, IL10/IL17, IL10/IL4, IFNγ/IL17, Th1/Th2, Th17/Th1, Th17/Th2, Th1/Treg, Th2/Treg, Th1 + Th2 + Th17/Treg, Th17/Treg, Th22/Th1, Th22/Th2, Th22/Th17, Th22/Treg and Th22 + Th17/Th1 + Th2. These ratios were calculated considering the T-cell response cytokines, either signature or induced cytokines (**Figure 1**) and represented using boxplots. Cytokines were included in each ratio according to the review of Wan and Flavell with the addition of the Th22 subset (Wan and Flavell, 2009).

### Statistical Analyses

All experiments were performed in independent biological quintuplicates. Data distribution did not follow normality, so initial comparisons were performed with the non-parametric Wilcoxon and Tukey pairwise tests. Differences in the value ranks between two conditions were assessed with the Mann–Whitney *U* tests for equal medians with Monte Carlo permutations (*n* = 99,999). Comparisons with a *p*-value ≤ 0.05 were considered statistically significant. Whisker and boxes plots were generated using ggplot2 in R environment, Past3 v3.15 (Hammer et al., 2001) and the IBM SPSS Statistics package (SPSS Inc., Chicago, IL, United States).

## Results and Discussion

In the present work we have synthesized 15 peptides (**Table 1**) encrypted in proteins produced by the human gut microbiome and selected due to their high potential immune modulatory properties predicted through the MAHMI pipeline^3^. To determine the immune response elicited by these bacterial peptides we selected an *in vitro* PBMC model from human healthy donors, as monocyte-derived dendritic cells have been shown as not suitable for these purposes (unpublished data). Eighteen cytokines related to T-cell response were quantified in the PBMC supernatants of five healthy donors after 5 days of co-culture with the peptides. Cytokine levels were measured basally (PBMCs without anti-CD3), in the activation control (PBMCs with anti-CD3), in the positive control (LPS + activated PBMCs) and for each peptide of study (peptide + activated PBMCs) (**Supplementary Figure S1**).

**FIGURE 2:**
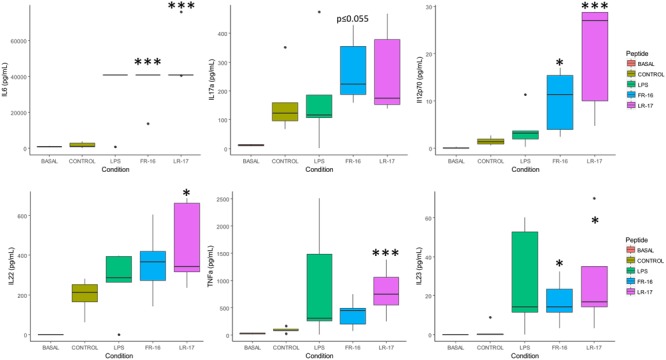
Main cytokine levels affected by the presence of peptides LR17 and FR16. Boxplots represent median and interquartile range for the selected cytokines (see all in **Supplementary Figure S1**). These were quantified in the supernatants after 5 days of *in vitro* co-culture of human PBMCs and the bacterial peptides FR-16 and LR-17, from *B. longum* DJO10A and *B. fragilis* YCH46, respectively. Significant differences were assessed by the non-parametric Mann–Whitney *U* test and are represented with ^∗^, ^∗∗^, or ^∗∗∗^ (*p* < 0.05, 0.01, or 0.001 respectively). Significant differences refer to control conditions (PBMCs + anti-CD3).

In general every peptide was sensed by PBMCs, which reacted with changes in their secreted cytokine profiles compared to the activation control. All the peptides displayed an effect modulating the production of at least one cytokine, although few of them induced consistently the production of interleukins from single T-cell response pathways, represented in **Figure 1**. For instance, the production of IL-2 and IL-6 were statistically induced (*p* < 0.05) by peptides FR-16, LR-17, AR-15, EY-12, KE-13 when compared to the control, as well as the LPS (**Supplementary Figure S1**). Peptides FR-16 and LR-17, with an immunomodulatory bioactivity prediction in MAHMI database of 100 and 80% respectively (**Table 1**), showed the higher immune modulation capability as reflected in the cytokine secretion induced over PBMCs (**Figure 2**).

FR-16 and LR-17 are bacterial peptides encrypted in proteins from *Bifidobacterium longum* DJ010A and *Bacteroides fragilis* YCH46, species that are amongst the more abundant members of the human microbiome (Arboleya et al., 2016; Lloyd-Price et al., 2016). As shown in **Figure 2**, both peptides increased the production of IL-6, IL17a, IL12p70, IL22, IL23 and TNFα either significantly or at the same levels than LPS did (with *p*-values very close to 0.05). All these cytokines are important in the Th17 and Th22 differentiation pathways (Wan and Flavell, 2009).

**FIGURE 3:**
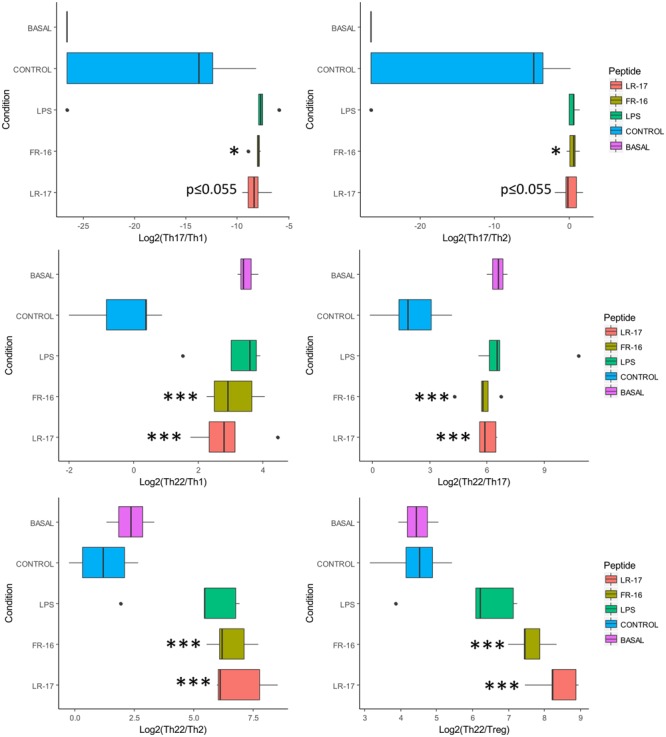
Ratios affected by the presence of peptides LR17 and FR16. Boxplots represent median and interquartile range for the ratios (see all in **Supplementary Figure S2**). Significant differences were assessed by the non-parametric Mann–Whitney *U* test and are represented with ^∗^, ^∗∗^, or ^∗∗∗^ (*p* < 0.05, 0.01, or 0.001 respectively). Significant differences refer to control conditions (PBMCs + anti-CD3).

As the T cell response in our model seemed to be modulated by FR16 and LR17, different ratios were calculated taken into account the inducing and signature cytokines for each pathway and also key cytokines such as IL10, TNFα or IFNγ (**Supplementary Figure S2**). As it can be shown in **Figure 3**, the Th17/Th1 and the Th17/Th2 ratio was consistently higher for both peptides compared with the anti-CD3 activation control. When Th22/Th1 and Th22/Th17 ratios were computed, the basal conditions were displaced toward the Th22 pathway and addition of the anti-CD3 antibody induced production of both Th1 and Th17 cytokines. Interestingly, increase in the Th22 pathway was significantly higher for both peptides whatever the pathway used for calculate the ratio, and always compared to the activation control.

Th17 and Th22 response develop important roles in the maintenance of gut homeostasis. In general, it has been reported that commensal microbiota influence the balance between Th1/Th2 (Ventura et al., 2012). Some probiotic bacteria possess immunomodulatory properties either by acting over immune cell populations and epithelial cells. This is the case of the yogurt starter *Lactobacillus delbrueckii* subsp. *bulgaricus* 8481 which reduced the concentration of cytokine IL-8 in serum following yogurt consumption. IL8 is an important pro-inflammatory chemokine produced by epithelial cells (and also macrophages) with induces chemotaxis in many immune cells, attracting them to infection sites (Moro-Garcia et al., 2013).

In bifidobacteria, the capability to induce different immune responses is a strain-dependent trait (Lopez et al., 2010), and for instance *B. bifidum* LMG13195 is able to induce Treg response (Lopez et al., 2011). On the other hand, surface proteins like the sortase-dependent pili from *B. bifidum* PRL2010 induced a Th1 response (Turroni et al., 2013). Other surface-associated protein are more related in regulating inflammatory responses such as *Lactobacillus acidophilus* S-layer A, which has been shown to protect mouse against experimentally induced colitis through a specific interaction with SIGNR3 (Lightfoot et al., 2015). Treg cells have become one of the main focuses of study in immune modulation properties of probiotics, and the balance between Treg/Th responses plays a key role in the maintenance of gut homeostasis (Maloy and Powrie, 2011). Treg induction has been previously demonstrated for where the surface associated proteins may play a key role. In our study, no significant differences were found regarding Treg cell differentiation induction by our selected peptides. However, interleukin 2 was upregulated by peptides FR-16 and LR-17 (and other peptides) (**Supplementary Figure S1**). IL-2 is directly related with Treg proliferation and maintenance of the subset population, and indeed Treg cells express the receptor for IL-2 (which is CD25) (Boyman and Sprent, 2012).

Expansion of Th17 cells is characteristic of segmented filamentous bacteria, which inhabits the murine gut and whose presence is directly linked with this specific T cell response (Ivanov et al., 2009; Farkas et al., 2015). Indeed presence of these bacteria confers resistance to the intestinal pathogen *Citrobacter rodentium* (Ivanov et al., 2009). There is strong scientific evidence of the key role of the gut microbiota in Th17 differentiation to promote a balance in the immune response to ensure a healthy status (Ivanov et al., 2008). In general, the Th17 pathway serves as protection against extracellular pathogens through recruitment of neutrophils (IL-17) and induction of anti-microbial peptides (IL-22). Th17 cells contribute to the resistance and the clearance of different enteropathogens such as *Salmonella* or *Listeria*, but also for airways pathogens such as *Klebsiella pneumoniae* (Happel et al., 2005; Kleinschek et al., 2006).

IL-22 is the signature cytokine of the Th22 cell subset and it has been related with the maintenance of the intestinal epithelium (tissue regeneration and cell proliferation) and contributing to the mucosal integrity ensuring the intestinal barrier function against pathogens (Rutz et al., 2014). It has also a role regulating CD4+ T cell response to commensal bacteria (Parks et al., 2016). One of the main roles of Th22 cells is to keep the epithelial barrier and reinforce it against potential pathogens such as *Clostridium difficile* (Hasegawa et al., 2014). IL-22 develops also important roles in intestinal epithelial cell proliferation and survival, two factors that are compromised in the course of an infection, and IL-22 is mainly produced by Th17 cells, Innate Lymphoid Cells, γδ T cells, and Natural Killer T cells (Ouyang et al., 2011). Human Th22 cells are characterized by producing low amounts of IL-17 and differenciate from naïve CD4+ T cells with IL-6 and without TGF-β (Zheng et al., 2007).

**FIGURE 4:**
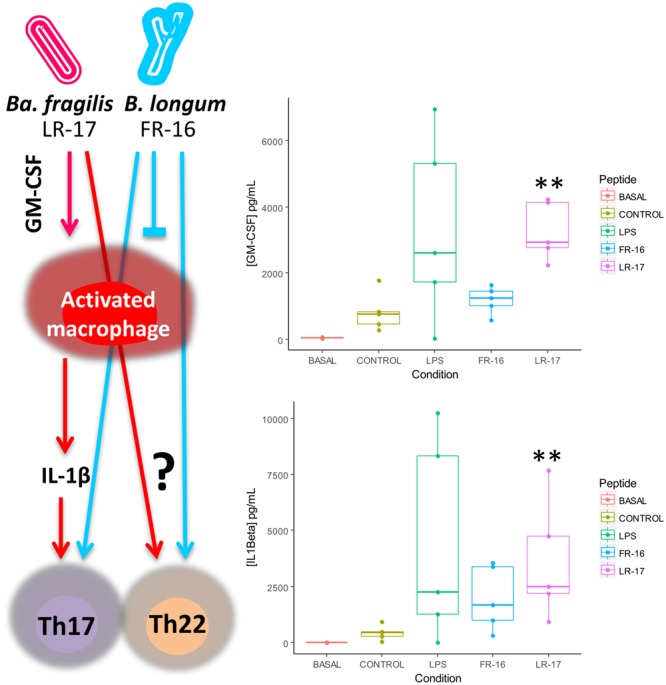
Proposed mechanism of action of LR17 and FR16 over the human immune system as supported by cytokine measurement. Significant differences were assessed by the non-parametric Mann–Whitney *U* test (^∗∗^*p*< 0.01). Significant differences refer to control conditions (PBMCs + anti-CD3).

LR-17 but not FR-16 induced production of GM-CSF and IL-1β, very similar to the action of LPS over PBMCs (**Figure 4**). These suggests that LR-17 action could be related to macrophage activation, which are important cells in inflammation with roles including elimination of pathogens and debris clearance. Macrophages show high degree of functional plasticity and may acquire different roles depending on the activation signals. It is well known that IFNγ-activated macrophages (Th1) are pro-inflammatory and antimicrobial, and their dysregulation is key in development of several inflammatory disorders, whereas IL4/IL13-activated macrophages (Th2) have anti-inflammatory roles (Arnold et al., 2015). Macrophages are very abundant in the inflammation sites, and indeed their numbers correlate with Th17 activity, contrarily to other antigen presenting cells such as dendritic cells (Allam et al., 2011). High amounts of IL-1β were produced in the presence of LR-17, but not in the presence of FR-16. IL-1β is produced by activated macrophages and is an important mediator of the inflammatory response and for Th17 expansion (Lasiglie et al., 2011), supporting Th17 expansion through macrophage activation as the mechanism of action of LR-17. In fact, the higher concentration of IL-1β induced by LR-17 correlates with the higher amounts of IL-22 detected, as the former induce Th17 cells to produce IL-22 (Monteiro et al., 2013). Finally this suggests the existence of a specific LR-17 receptor on antigen presentation cells, although this hypothesis deserves further research.

Why LR-17 and not FR-16 activated macrophages is out of the scope of our experimental setup. Strains of the species *Bacteroides fragilis* are known for the production of Polysaccharide A (PSA), an extracellular glycan with anti-inflammatory properties (Mazmanian et al., 2008), but many other surface components have been shown to possess immunomodulatory activity (Wexler and Goodman, 2017). On the contrary, higher abundances of commensal bifidobacteria are linked to Treg promoting pathways (Lopez et al., 2012), and many of the probiotic products targeting intestinal inflammatory disorders contain bifidobacteria (Ng et al., 2010). The cellular type targeted by bifidobacterial FR-16 peptide remains a mystery.

It must be considered that dysregulation of both Th17 and Th22 pathways are linked to inflammatory and autoimmune diseases. For instance higher production of IL-22 in the colonic mucosa has been observed in patients suffering from inflammatory bowel disease (Parks et al., 2016), and a pathogenic role for the overproduction of this cytokine has been proposed in rheumatoid arthritis (Rutz et al., 2014). However, increasing the Th17 response may be protective in other autoimmune diseases such as type 1 diabetes, so this type of encrypted peptides could have an application in the remission of these disorders (Sanchez et al., 2015). In addition, *in vivo* studies suggest that infection by *Citrobacter rodentium* in IL-23 -/- mice can be prevented by transferring Th22 but not Th17 cells (Basu et al., 2012). Further research will elucidate precisely the mechanism of action of this type of peptides over CD4+ effector T cells.

## Conclusion

All bacterial peptides analyzed in this study were able to modulate, *in vitro*, the immune response of human PBMCs, based on the cytokine pattern production. These results corroborated the *in silico* prediction performed with MAHMI database showing the usefulness of this tool to make accurate prediction for a screening in the selection of bioactive peptides. Results obtained for these peptides using human PBMCs strongly suggested that these Th17-promoting peptides might be detected by specific receptors, although this is a speculative statement deserving further experiments. The peptides FR-16 and LR-17, encrypted within the human gut metaproteome, were able to induce a higher immune response that is related with Th17 and Th22 responses. Incubation of these peptides with PBMCs allowed us to test the hypothesis of whether our gut microbiota is able to interact with the immune system through peptides encrypted in larger proteins. We propose the possibility to use bacterial peptides encrypted in the human microbiome proteins, as key inductors to modulate the human immune response for several immunological disorders, as recently reviewed (Blanco-Míguez et al., 2016).

## Author Contributions

BS conceived the experiments and wrote the manuscript. RA-A and MM-G designed the immunological study, AB-M, FF-R, and AL participated in the design of the MAHMI database and peptide retrieval, and CH-C and MM-G performed the experiments and drafted the first version of the manuscript. All authors reviewed the final version of the manuscript.

## Conflict of Interest Statement

BS and CH-C are on the scientific board and co-founders of Microviable Therapeutics. The other authors declare that the research was conducted in the absence of any commercial or financial relationships that could be construed as a potential conflict of interest.

## Footnotes

1 http://www.mahmi.org

2 http://gigadb.org/dataset/100064

3 http://www.mahmi.org

## Supplementary Material

FIGURE S1 Boxplots represent median and interquartile range of cytokines (pg/mL) quantified in the supernatants after 5 days of *in vitro* co-culture of human PBMCs and bacterial peptides.

FIGURE S2 Ratios among the different cytokines taken into account the distribution depicted in **Figure 1**.
