# Supplementary figures and images for: In Silico Screening of the Human Gut Metaproteome Identifies Th17-Promoting Peptides Encrypted in Proteins of Commensal Bacteria
Source: Front Microbiol. 2017 Sep 8;8:1726. doi: 10.3389/fmicb.2017.01726 (PMC5596104; doi:10.3389/fmicb.2017.01726)

GM/CSF

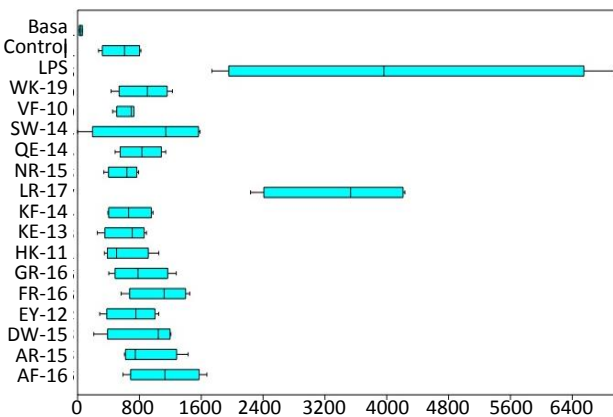IFN $\gamma$ 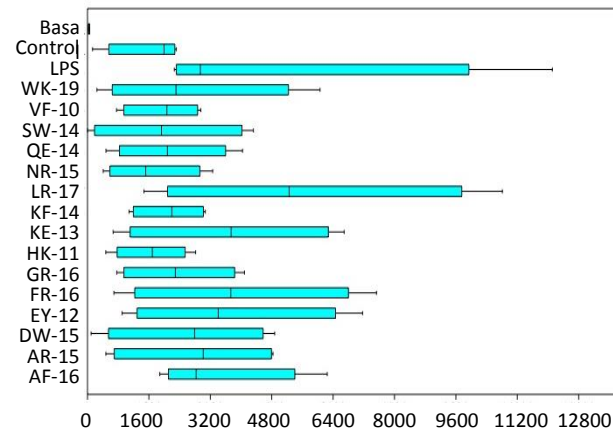

IL-10

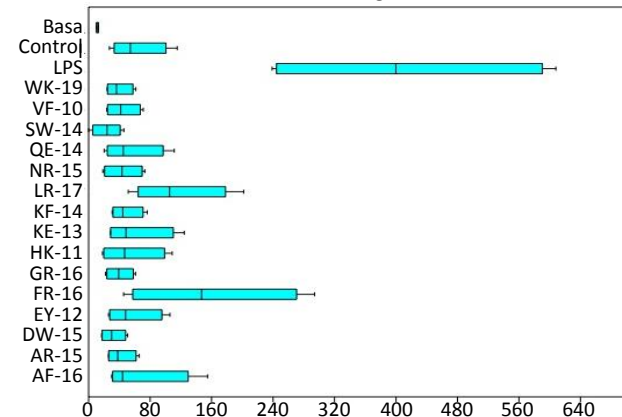

IL-12p70

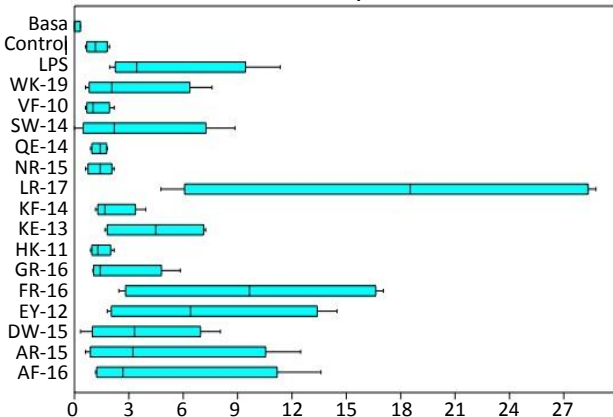

IL-13

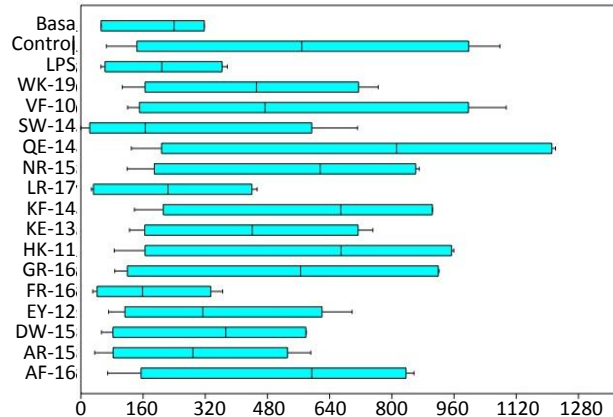

IL-17a

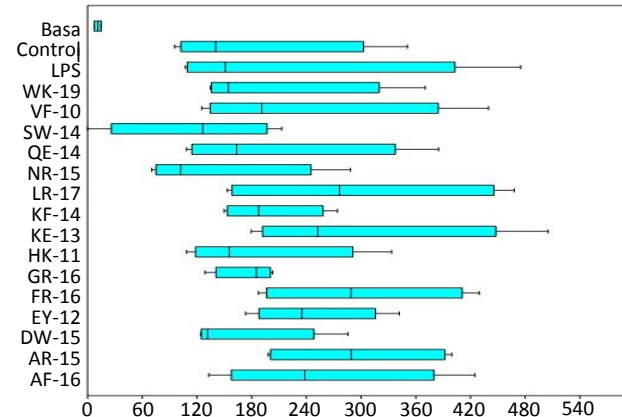

IL-18

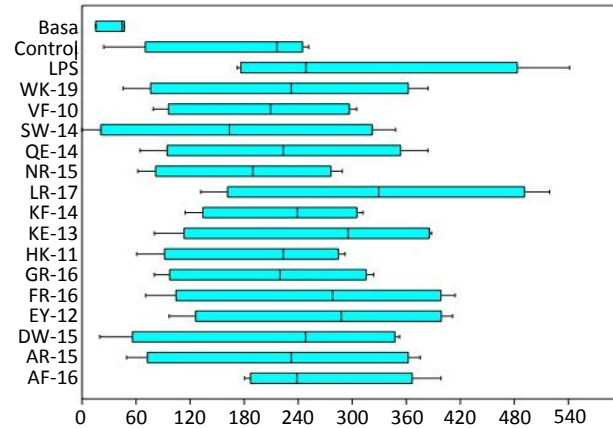IL-1 $\beta$ 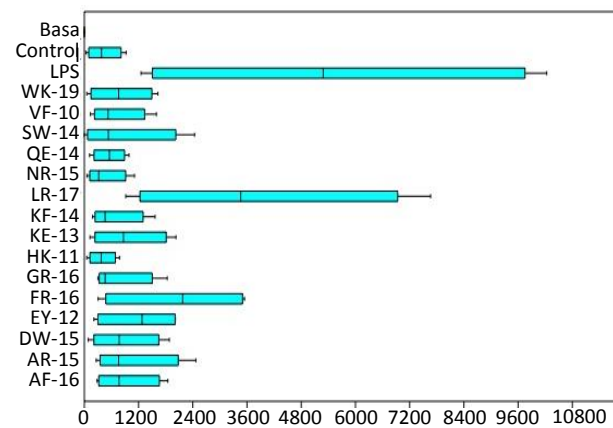

IL-2

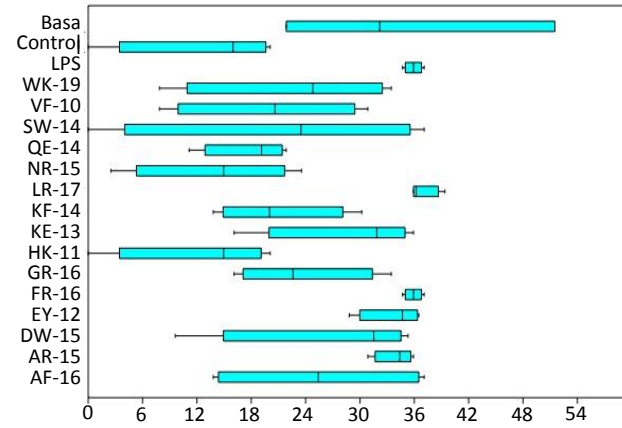

IL-21

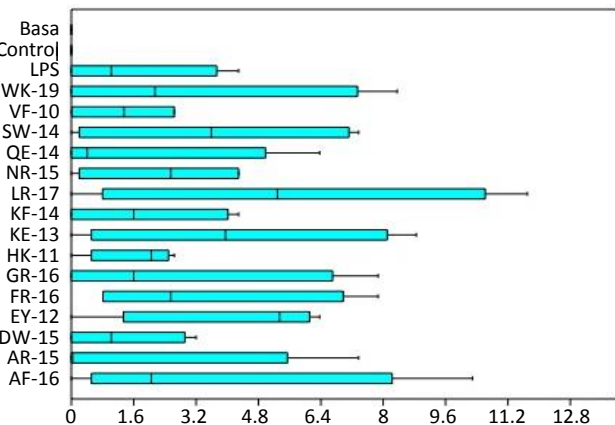

IL-22

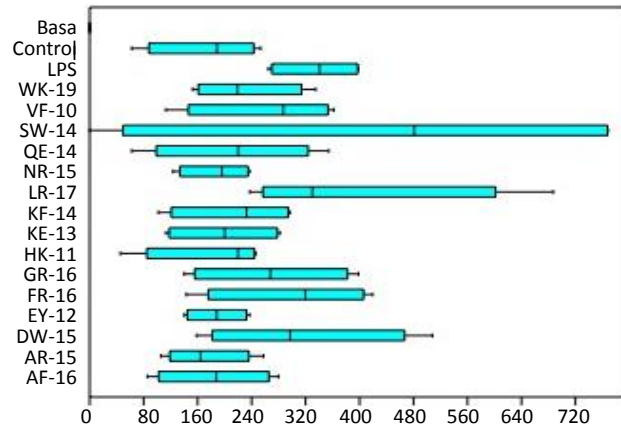

IL-23

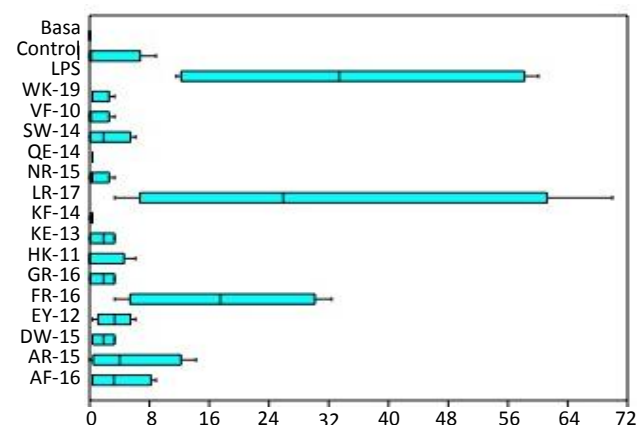

IL-27

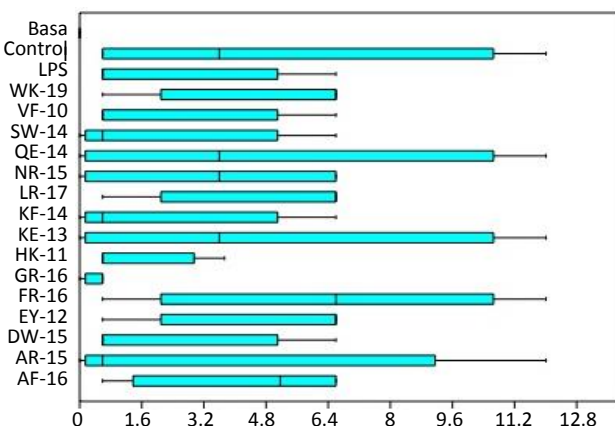

IL-4

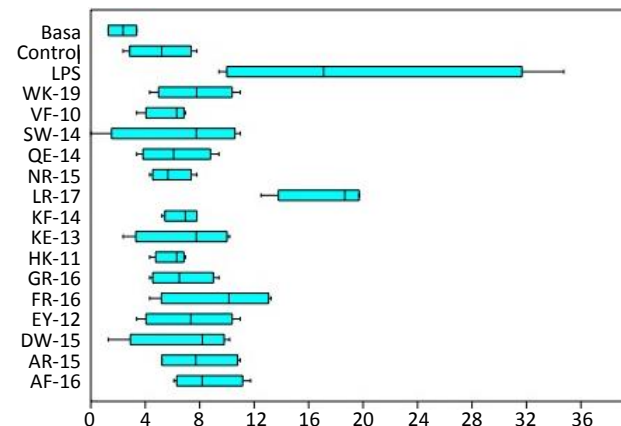

IL-5

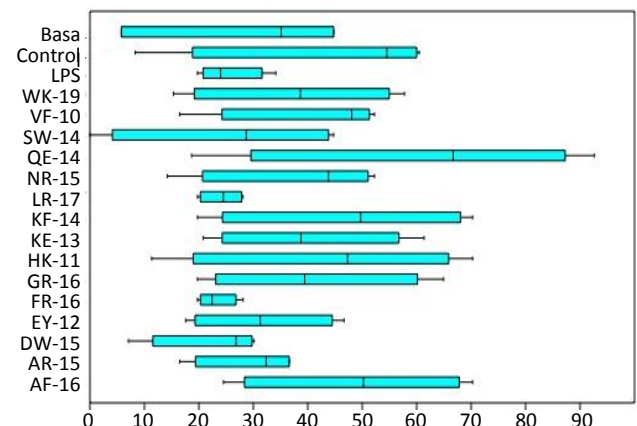

IL-6

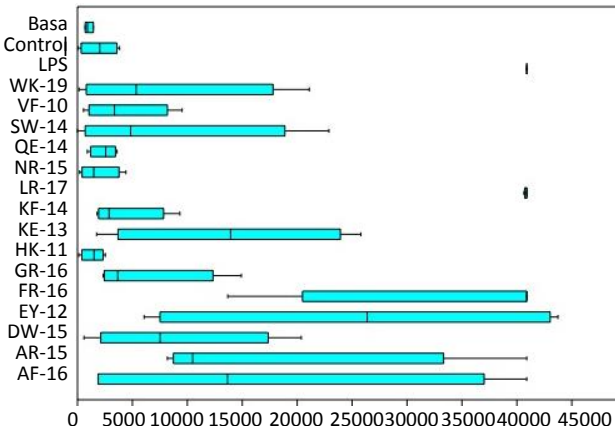

IL-9

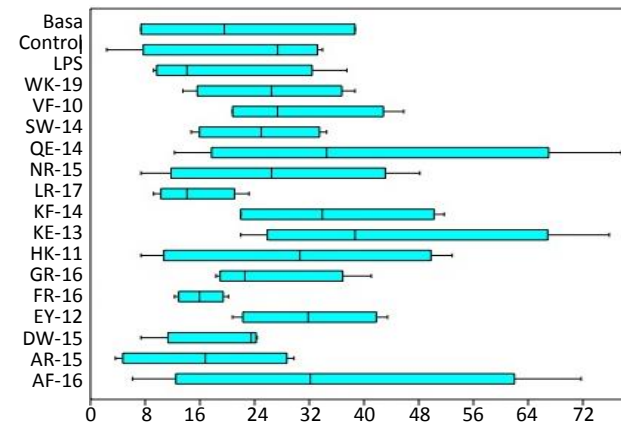

TNFα

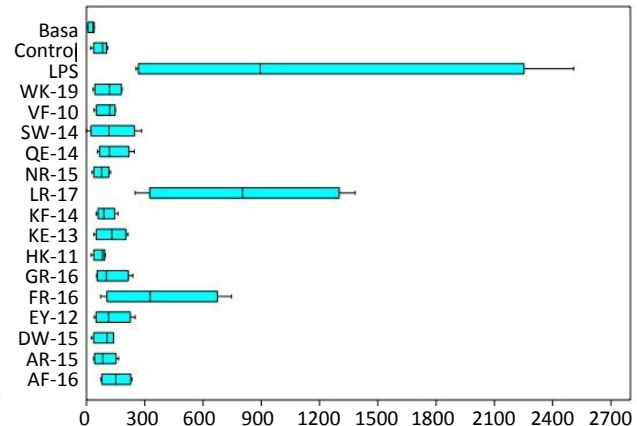

Supplement: FIGURE S1 — Boxplots represent median and interquartile range of cytokines (pg/mL) quantified in the supernatants after 5 days of in vitro co-culture of human PBMCs and bacterial peptides. [file Image_1.PDF]
