# Supplementary material for: In Silico Screening of the Human Gut Metaproteome Identifies Th17-Promoting Peptides Encrypted in Proteins of Commensal Bacteria
Source: Front Microbiol. 2017 Sep 8;8:1726. doi: 10.3389/fmicb.2017.01726 (PMC5596104; doi:10.3389/fmicb.2017.01726)

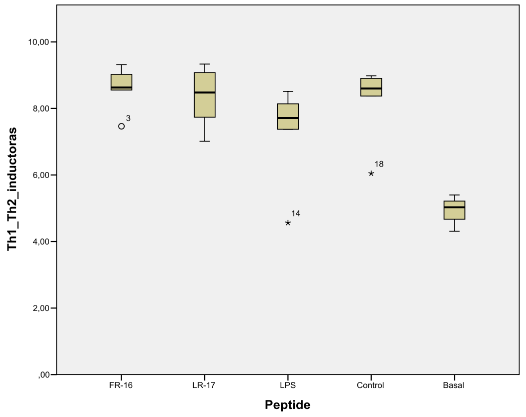

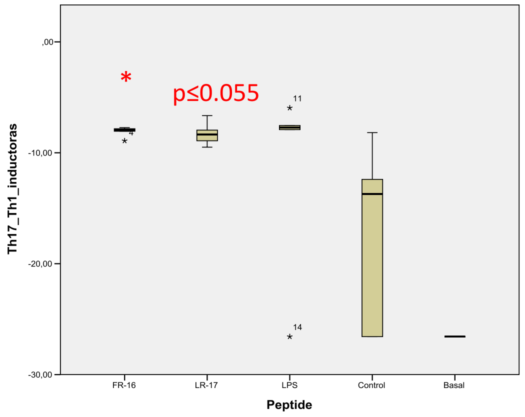

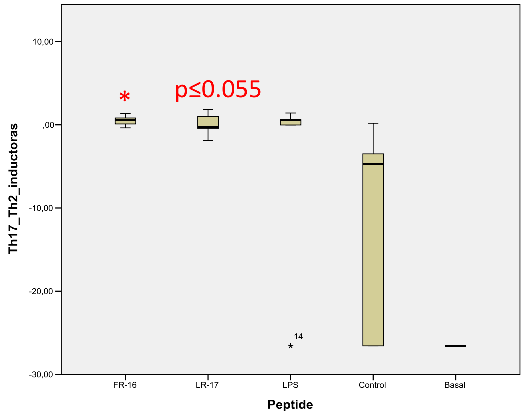

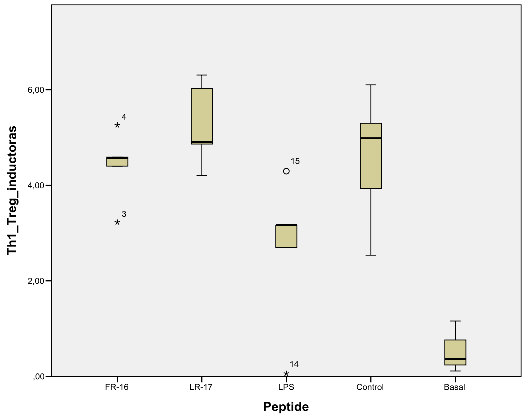

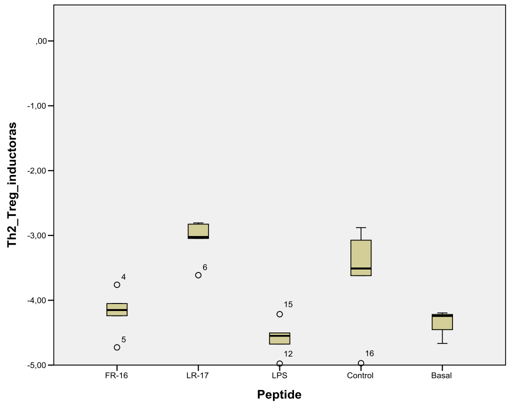

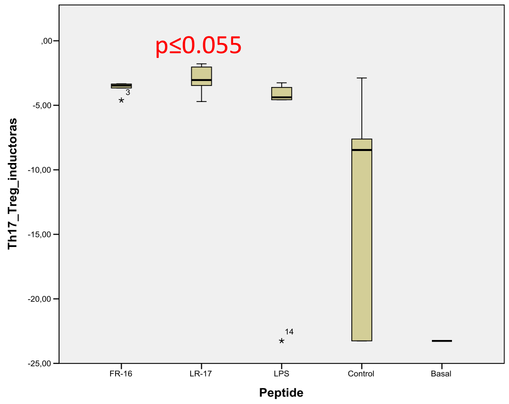

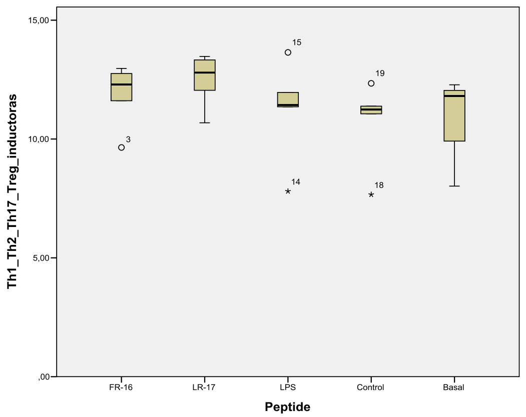

Slide 1: Ratios calculated with inducing cytokines

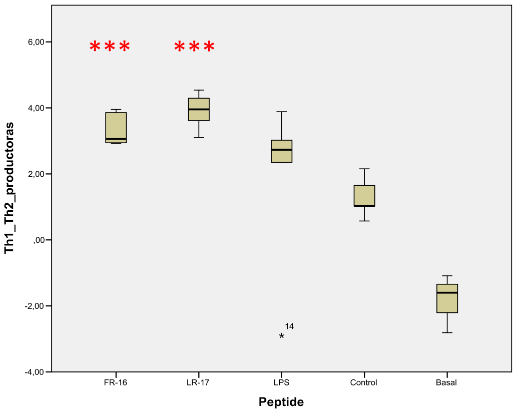

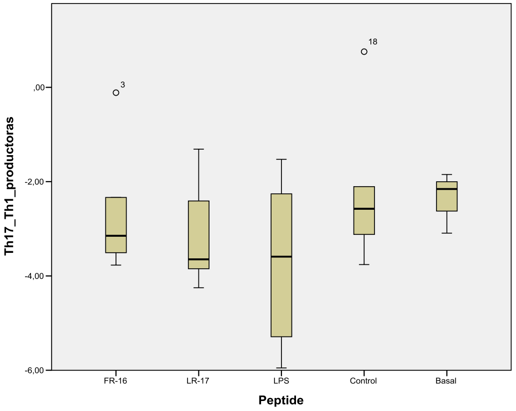

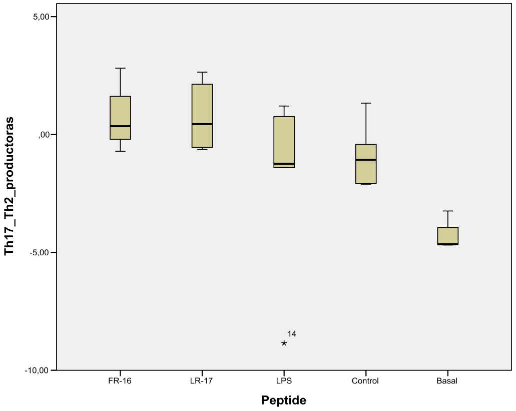

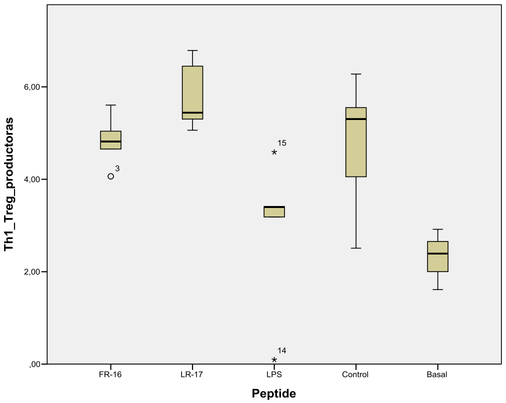

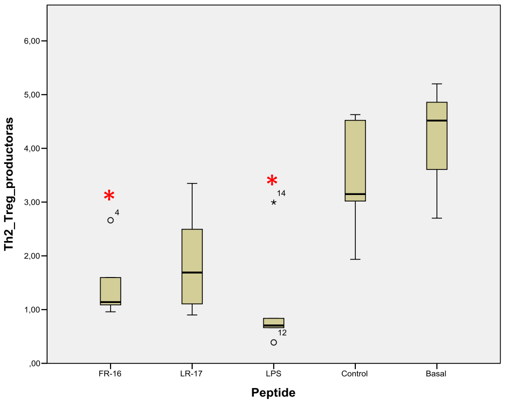

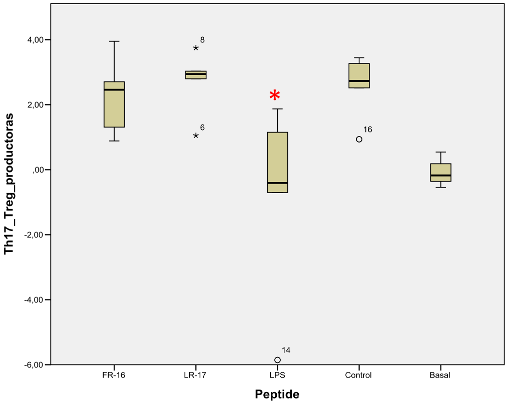

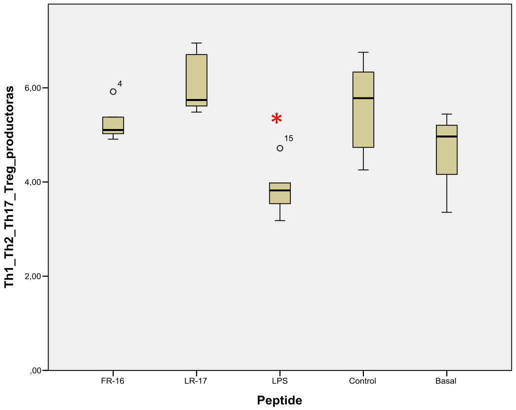

Slide 2: Ratios calculated using signature cytokines

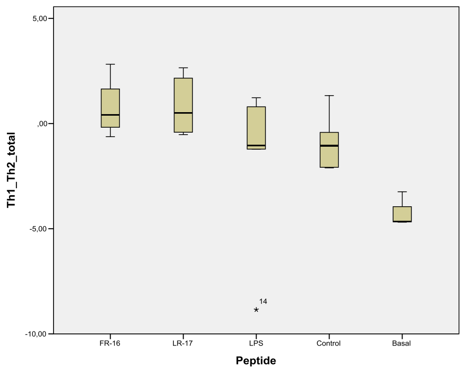

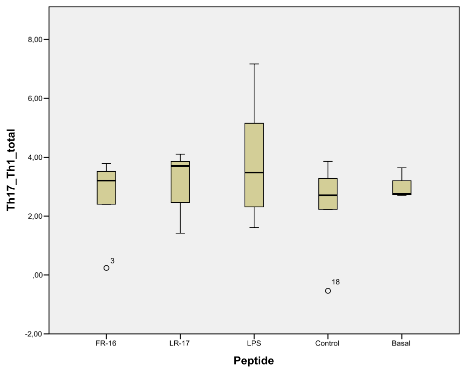

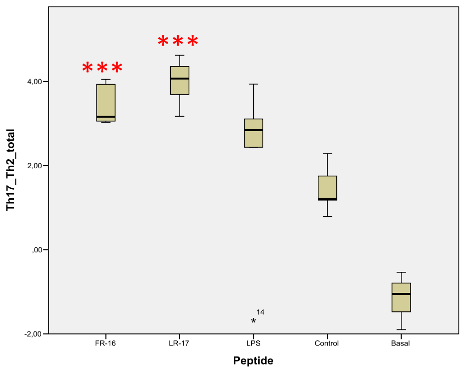

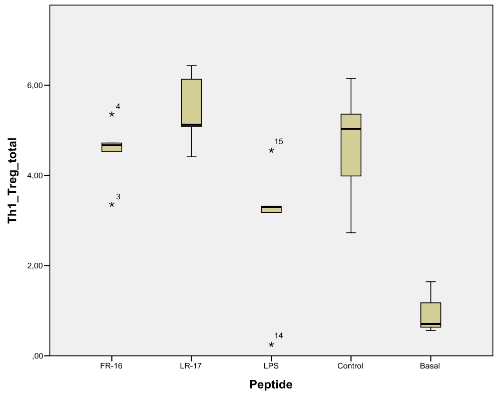

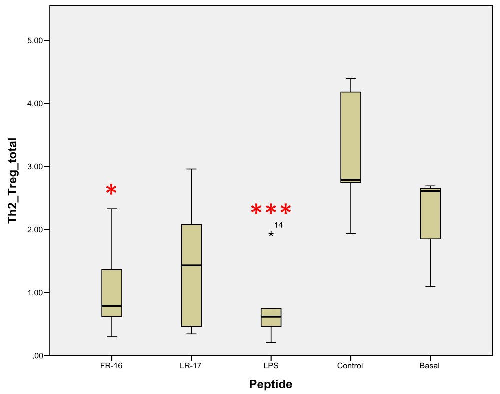

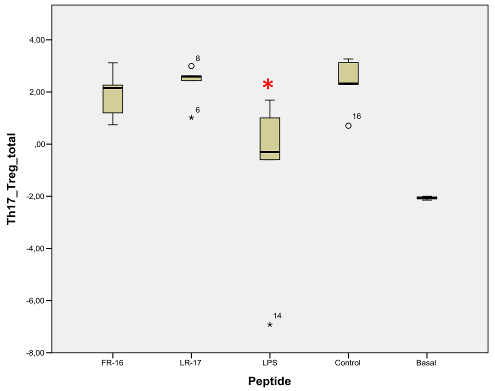

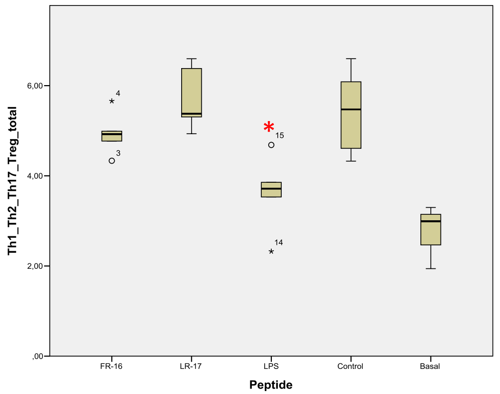

Slide 3: Ratios calculated using total cytokines

Slide 4: Ratios calculated using key cytokines

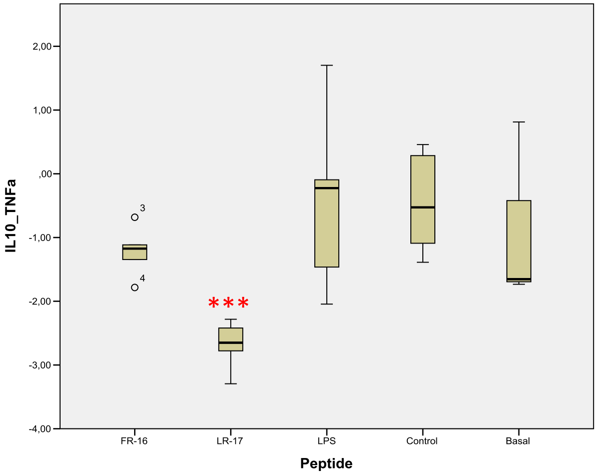

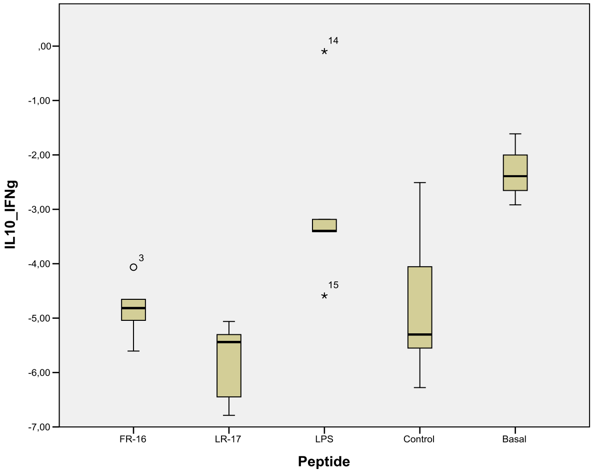

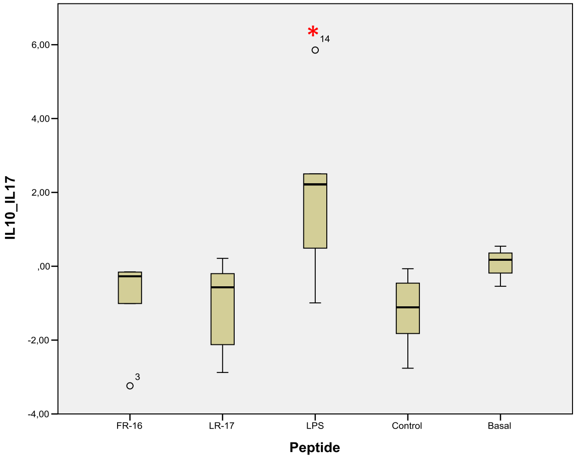

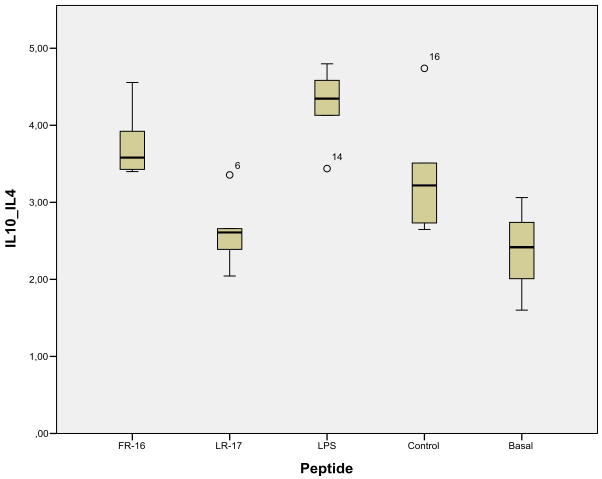

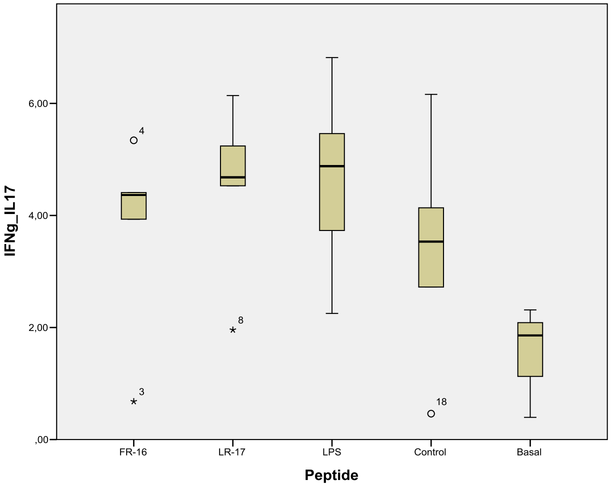

Supplement: FIGURE S2 — Ratios among the different cytokines taken into account the distribution depicted in Figure 1. [file Image_2.PDF]
